# Neuropsychiatric symptoms with focus on apathy and irritability in sporadic and hereditary cerebral amyloid angiopathy

**DOI:** 10.1186/s13195-024-01445-4

**Published:** 2024-04-06

**Authors:** Kanishk Kaushik, Anna M. de Kort, Rosemarie van Dort, Reinier G.J. van der Zwet, Bob Siegerink, Sabine Voigt, Erik W. van Zwet, Maaike C. van der Plas, Emma A. Koemans, Ingeborg Rasing, Roy P.C. Kessels, Huub A.M. Middelkoop, Floris H.B.M. Schreuder, Catharina J.M. Klijn, Marcel M. Verbeek, Gisela M. Terwindt, Ellis S. van Etten, Marieke J.H. Wermer

**Affiliations:** 1 Neurology, Leiden University Medical Center (LUMC), Albinusdreef 2, 2300RC, Leiden, NL the Netherlands; 2 Neurology, Radboud University Medical Center (RUMC), Nijmegen, the Netherlands; 3 Donders Institute for Brain, Cognition and Behavior, Radboud University, Nijmegen, the Netherlands; 4 Clinical Epidemiology, LUMC, Leiden, the Netherlands; 5 Radiology, LUMC, Leiden, the Netherlands; 6 Biomedical Data Sciences, LUMC, Leiden, the Netherlands; 7 Medical Psychology and RUMC Alzheimer Center, Nijmegen, the Netherlands; 8 Vincent van Gogh Institute for Psychiatry, Venray, the Netherlands; 9 Institute of Psychology, Health and Neuropsychology, Leiden University, Leiden, the Netherlands; 10 Laboratory Medicine, RUMC, Nijmegen, the Netherlands; 11 Neurology, University Medical Center Groningen, Groningen, the Netherlands

**Keywords:** Cerebral amyloid angiopathy, CAA, Neuropsychiatric symptoms, ICH, Apathy, Irritability, Depression, Executive function, Processing speed, Cognitive decline

## Abstract

**Background:**

Neuropsychiatric symptoms (NPS) may affect cognition, but their burden in cerebral amyloid angiopathy (CAA), one of the main causes of intracerebral hemorrhage (ICH) and dementia in the elderly, remains unclear. We investigated NPS, with emphasis on apathy and irritability in sporadic (sCAA) and Dutch-type hereditary (D-)CAA.

**Methods:**

We included patients with sCAA and (pre)symptomatic D-CAA, and controls from four prospective cohort studies. We assessed NPS per group, stratified for history of ICH, using the informant-based Neuropsychiatric Inventory (NPI-Q), Starkstein Apathy scale (SAS), and Irritability Scale. We modeled the association of NPS with disease status, executive function, processing speed, and CAA-burden score on MRI and investigated sex-differences.

**Results:**

We included 181 participants: 82 with sCAA (mean[SD] age 72[6] years, 44% women, 28% previous ICH), 56 with D-CAA (52[11] years, 54% women, *n* = 31[55%] presymptomatic), and 43 controls (69[9] years, 44% women). The NPI-Q NPS-count differed between patients and controls (sCAA-ICH+:adj.β = 1.4[95%CI:0.6–2.3]; sCAA-ICH-:1.3[0.6-2.0]; symptomatic D-CAA:2.0[1.1–2.9]; presymptomatic D-CAA:1.2[0.1–2.2], control median:0[IQR:0–3]), but not between the different CAA-subgroups. Apathy and irritability were reported most frequently: *n* = 12[31%] sCAA, 19[37%] D-CAA had a high SAS-score; *n* = 12[29%] sCAA, 14[27%] D-CAA had a high Irritability Scale score. NPS-count was associated with decreased processing speed (adj.β=-0.6[95%CI:-0.8;-0.4]) and executive function (adj.β=-0.4[95%CI:-0.6;-0.1]), but not with radiological CAA-burden. Men had NPS more often than women.

**Discussion:**

According to informants, one third to half of patients with CAA have NPS, mostly apathy, even in presymptomatic D-CAA and possibly with increased susceptibility in men. Neurologists should inform patients and caregivers of these disease consequences and treat or refer patients with NPS appropriately.

**Supplementary Information:**

The online version contains supplementary material available at 10.1186/s13195-024-01445-4.

## Background

Neuropsychiatric symptoms (NPS), specifically apathy, are common in Alzheimer’s disease (AD), vascular dementia and cerebral small-vessel disease (CSVD) [1, 2]. These symptoms may affect the rate of cognitive decline and quality of life, and might vary by sex [3–5]. Cerebral amyloid angiopathy (CAA) is a common CSVD characterized by accumulation of amyloid-β in cerebral vessels. Patients can present with lobar intracerebral hemorrhage (ICH), cognitive impairment, or transient focal neurological episodes (TFNE).

NPS can occur in CAA, but little is known about their frequency or possible sex-differences [6]. At conception of the current investigation, no NPS-related CAA-literature existed. Since then, one neuropathological study found overlap in the NPS-profiles of CAA and AD [1]. Another study found depression, irritability, agitation, and apathy to be prevalent in one third to half of patients [7]. In a third study, a higher degree of apathy correlated with higher CAA-burden on MRI [8]. This was similar to a study in patients with ICH where apathy and hyperactivity (agitation, irritability, disinhibition) were linked to a CAA-MRI profile [9]. The recognition of NPS is important, as they are sometimes treatable. Replication of these findings is warranted because the sample sizes and underrepresentation of patients with history of ICH in these studies limits their generalizability.

The prevalent non-hereditary sporadic form of CAA (sCAA) by definition is only present in patients ≥ 50 years. However, genetic types like Dutch-type CAA (D-CAA) present at younger ages [10]. D-CAA is caused by a point-mutation in the amyloid precursor protein (APP)-gene. D-CAA has a similar, but accelerated disease course with an onset approximately 20 years earlier than sCAA [11, 12]. Therefore, in contrast to sCAA, D-CAA has limited age-related pathology, making it an excellent model for studying the disease consequences. Given the similarities of sCAA and (pre)symptomatic D-CAA, we expect their NPS-profiles to be similar. However, to date no systematic research on this topic has been performed [11]. 

Impaired executive functioning and processing speed associated with CAA might contribute to developing NPS like apathy and irritability, possibly due to disruption of the orbitofrontal circuits, or cognitive overload caused by progressive cognitive decline [12–14]. We expect these symptoms to be prevalent and linked to cognitive decline or CAA-burden on MRI.

Therefore, we aim to describe (i) the neuropsychiatric profile in patients with sCAA and D-CAA with emphasis on the frequency of apathy and irritability, and attention to the presymptomatic phase of D-CAA, using informant-based questionnaires. We assessed (ii) the association of NPS with cognitive functioning in the domains of executive function and processing speed, as well as with (iii) the global and frontal CAA disease burden on MRI (CAA-burden). We (iv) assessed sex-differences for developing NPS.

## Methods

### Participants

We included participants from four ongoing prospective cohorts (all started 2018): patients with sCAA from the Follow-up in sporadic CAA study (FOCAS) and from the BIOmarkers for cogNitive Impairment due to CAA study (BIONIC); patients with D-CAA from the HCHWA-D follow-up study (AURORA); and controls from FOCAS, AURORA, and the CAA Fluid Biomarkers Evaluation study (CAFE) [15]. The local ethics review boards approved all studies (AURORA: NL62670.058.17; BIONIC: 2017–3810; FOCAS: NL63256.058.17). All participants provided written informed consent.

Patients with sCAA were all diagnosed as ‘probable CAA’ according to the modified Boston criteria [16]. Patients with sCAA and previous symptomatic ICH were included in BIONIC at > 3 months after the ICH. We included (pre)symptomatic participants with D-CAA who were aged ≥ 18 years and had either (i) a DNA-proven mutation of codon 693 of the APP-gene, or (ii) a medical history of ≥ 1 symptomatic ICH with ≥ 1 first-degree relative with DNA-confirmed D-CAA. Patients with D-CAA with previous symptomatic ICH were considered symptomatic.

The control group, which is intended to function as a reference for the NPS-profile in absence of CAA or (subjective) cognitive decline, was selected to be cognitively healthy and free of CAA: those from FOCAS, recruited through newspaper advertising, were all without history of brain disease and without (subjective) substantial memory complaints; those from AURORA had DNA-proven absence of the APP-gene mutation; those from CAFE, recruited from the Dutch Brain Research Registry, were partners and relatives of patients without (subjective) memory complaints, history of brain- or neurodegenerative disease, and with intact global cognition in cognitive screening (Supplemental Methods; Figure S1) [17, 18] Controls were excluded if they had clinical symptoms of CAA or if they met the modified Boston Criteria for probable or possible CAA on their MRI. Controls were not explicitly screened for presence of other CSVD on MRI but all were known not to have a formal previous diagnosis of brain- or neurodegenerative disease, and did not have any clinical complaints.

### Data collection

#### Clinical data

We collected all clinical data, neuropsychiatric questionnaires, neuropsychological test results and MRI at one study assessment. Data on demographics, medical history, and clinical symptoms including history of symptomatic ICH were prospectively obtained in standardized annual study visits. Time since previous symptomatic ICH at the date of study assessment was obtained from electronic patient files. Current depressive episodes were assessed with the Center for Epidemiological Studies Depression scale (CES-D; cut-off ≥ 16), combined with self-reported history of depression during the study visit (AURORA/FOCAS only) [19]. Educational level was categorized (low/average/high) according to the standardized Dutch classification [20]. 

#### Neuropsychiatric questionnaires

We measured NPS during the month preceding the study visit with the 12-item informant-based Neuropsychiatric Inventory–Questionnaire (NPI-Q; Dutch translation; all source cohorts), assessing: agitation, anxiety, apathy, appetite, delusions, depression, disinhibition, euphoria, hallucinations, irritability, motor disturbances and night-time behaviors. The informant (first-degree relative) first answers a screening question (absent/present) per symptom-domain. If present, symptom severity (mild/moderate/severe) and caregiver burden (6-point scale ranging from “not at all” to “extreme”) are rated. The NPI-Q total (NPS-count) ranges from 0 to 12, severity from 0 to 36, and caregiver burden from 0 to 60 [21]. 

To improve accuracy, apathy and irritability were assessed in twofold. Besides the NPI-Q, we administered Dutch versions of two validated informant-based instruments: the Starkstein Apathy scale (SAS) and the Irritability Scale. Scores on the 14-item SAS range between 0 and 42, with a score ≥ 14 being indicative of apathy [4]. Similarly, the 14-item Irritability Scale scores 0 (absent) to 3 (maximum intensity of behavior) per question (range 0–42; cut-off ≥ 14; AURORA/FOCAS only) [22]. 

Participants were included, if at least one NPI-Q or SAS was completed in the respective source cohort before March 2023, and excluded in case of non-response or non-consent. If multiple visits with questionnaires were available, the first visit with the most complete data was used.

#### Neuropsychological testing

Trained researchers administered a standardized 90-minute neuropsychological assessment using widely recognized, validated and translated instruments in all source-cohorts. To ensure similar phenotyping and data compatibility, we established comparable testing-protocols at cohort conception. General cognition was assessed with the Montreal Cognitive Assessment (MoCA) version 7.1 [23]. We assessed executive function and processing speed through the Stroop Color-Word Test, Trail Making Test (TMT), category verbal fluency (animal naming), Frontal Assessment Battery (FAB; AURORA/FOCAS only), and the Symbol Digit Substitution Task (SDMT; BIONIC/CAFE only) [24–28]. We assessed memory with the Rey Auditory-Verbal Learning-test [29]. 

#### CAA-burden on MRI

Two experienced independent raters graded presence of CAA-related markers for patients and controls on 3.0 Tesla MRI according to the STandards for ReportIng changes on nEuroimaging (STRIVE) and previously described rating scales (Supplemental Methods) [16, 30–32]. The CAA-burden score summarizes lobar cerebral microbleeds (CMB), cortical superficial siderosis (cSS), white matter hyperintensities (WMH) and centrum semi-ovale enlarged perivascular spaces (CSO-EPVS) in a 6-point ordinal scale (Supplemental Methods) [33]. We defined frontal CAA-burden as the number of frontal CMBs and absence/presence of cSS.

### Statistical analysis

Data are presented for patients with sCAA (all and stratified for history of ICH), D-CAA (all and stratified for history of ICH), and controls separately. Demographic data, clinical characteristics, and frequencies are displayed as means with standard deviations (SD), median with interquartile ranges (IQR), or proportions (%) as appropriate. Median and dichotomous (cut-off ≥ 14) SAS-data are presented. We analyzed descriptive statistics for the NPI-Q symptom profile and severity scores. We describe frequencies of informant-reported apathy and irritability. We calculated odds ratios (OR) with 95% confidence interval (95%CI) of having (i) any NPS, (ii) apathy or (iii) irritability in patients with presymptomatic D-CAA compared to controls.

Cognitive flexibility was measured through the TMT B/A-ratio [34]. We calculated age, sex and education adjusted Z-scores for the individual neuropsychological tests, using normative data for the Dutch population, and averaged them into domain scores [35, 36]. For executive function, this encompasses cognitive flexibility and Stroop interference time (Stroop-III/II ratio). For processing speed, these were the TMT-A, Category fluency, Stroop-I and Stroop-II. Aborted tests were assigned Z-score − 3SD. A domain Z-score [-1.0;-1.5] was considered below average, and Z< -1.5SD as cognitive impairment [37]. We calculated unadjusted ORs for sex-differences in frequency of NPS, apathy, and irritability for all participants with CAA combined.

Because apathy might be associated with changes in Aβ burden in the cerebrospinal fluid (CSF) of patients with Alzheimer’s Disease, we assessed differences in CSF levels of Aβ-40, Aβ-42, tau and phosphorylated tau, between patients with and without apathy according to the NPI-Q with the Mann-Whitney U test (Supplemental Methods) [15, 38]. 

With linear regression modelling (ANCOVA) we assessed whether total NPI-Q score was associated with disease group category (sCAA-ICH+/sCAA-ICH-/symptomatic D-CAA/presymptomatic D-CAA), with controls as reference, controlling for age and sex. The same model was constructed for SAS-score. We constructed two separate models to assess the association between total NPI-Q score and (i) executive function and (ii) processing speed (both norm-adjusted Z-scores), controlling for disease group and time since previous symptomatic ICH. A fifth model was made to assess the relation of total NPI-Q score with total CAA-burden score, controlling for disease group, age, and sex. Finally, we modelled the association of NPI-Q score with frontal CAA-burden (number of CMBs and absence/presence of cSS entered separately into the model), controlling for the same confounders. To retain statistical power, depression was not entered in the models. We present adjusted coefficients (adj.β) and provide the unadjusted covariates in Appendix S1.

We compared patient characteristics (demographics, medical history, cognitive performance) of all included participants to those without ≥ 1 NPI-Q or SAS (recorded for AURORA/FOCAS only) in a descriptive sensitivity analysis. To analyze robustness, we performed another sensitivity analysis in only the BIONIC/CAFE participants, in which the processing speed Z-score was extended by the SDMT.

Analyses were performed using R (version 4.2.1) and α was set at 0.05. We corrected for multiple comparisons with Tukey’s HSD method. This manuscript follows the STROBE reporting guideline.

## Results

We included 181 participants: 82 with sCAA (mean age 72 years, 44% women, 28% history of ICH), 56 with D-CAA (mean age 52 years, 54% women, 25[45%] history of ICH), and 43 controls (mean age 69 years, 44% women, Table 1, Table S1).

**Table 1:** Baseline characteristics

	sCAA, all *82*	sCAA ICH + *23*	sCAA ICH- *59*	D-CAA, all *56*	D-CAA symptomatic *25*	D-CAA presymptomatic *31*	Control *43*
Age, years, mean(SD)	72 (6)	71 (6)	72 (6)	52 (11)	60 (8)	46 (10)	69 (9)
Men, *n*(%)	46 (56)	15 (65)	31 (53)	26 (46)	13 (52)	13 (42)	24 (56)
Educational level, n(%)							
Low	28 (34)	5 (22)	23 (39)	14 (25)	9 (36)	5 (16)	8 (19)
Average	21 (26)	8 (33)	13 (22)	20 (36)	5 (20)	15 (48)	11 (26)
High	27 (33)	8 (33)	19 (33)	16 (29)	8 (32)	8 (26)	21 (49)
MoCa, mean(SD)	24 (5)	25 (4)	24 (6)	27 (3)	26 (3)	27 (2)	27 (2)
Medical History, n(%)							
Symptomatic ICH*	23 (28)	23 (100)	-	25 (45)	25 (100)	-	-
Cognitive decline	33 (40)	7 (30)	26 (44)	13 (23)	9 (36)	4 (13)	-
Hypertension	44 (54)	12 (52)	32 (54)	13 (23)	6 (24)	7 (23)	19 (44)
Hypercholesterolemia	43 (52)	15 (65)	28 (48)	6 (11)	4 (16)	2 (7)	13 (30)
Diabetes Mellitus	5 (6)	1 (4)	4 (7)	2 (4)	1 (4)	1 (3)	5 (12)
Depression	3 (4)	0 (0)	3 (5)	8 (14)	3 (12)	5 (16)	1 (2)
Time since last ICH†, years, median[IQR]	2 [1,4]	2 [1,4]	-	3 [1,4]	3 [1,4]	-	-
Previous symptomatic ICH count, median[IQR]	0 [0,1]	1 [1,1]	-	0 [0,1]	1 [1,2]	-	-
ICH in limbic regions of the brain^§^, n(% of all ICH)	2 (6)	2 (6)	-	10 (23)	10 (23)	-	-

D-CAA Dutch-type cerebral amyloid angiopathy; sCAA sporadic CAA; ICH intracerebral hemorrhage; MoCA Montreal Cognitive Assessment

* Location % frontal/parietal/temporal/occipital: sCAA 38/29/15/21; D-CAA 30/21/21/35

§ Orbito-frontal cortex, cingulate cortex or anterior temporal lobe; Basal ganglia (ventral striatum), hippocampus, amygdala and fornix by definition not possible in CAA due to strictly lobar ICH

† Recorded only for patients with history of symptomatic ICH

Educational level missing in *n* = 2 sCAA-ICH+, *n* = 4 sCAA ICH-, *n* = 3 symptomatic D-CAA/presymptomatic D-CAA/controls

### Frequency of NPS, apathy, and Irritability

**Fig. 1:**
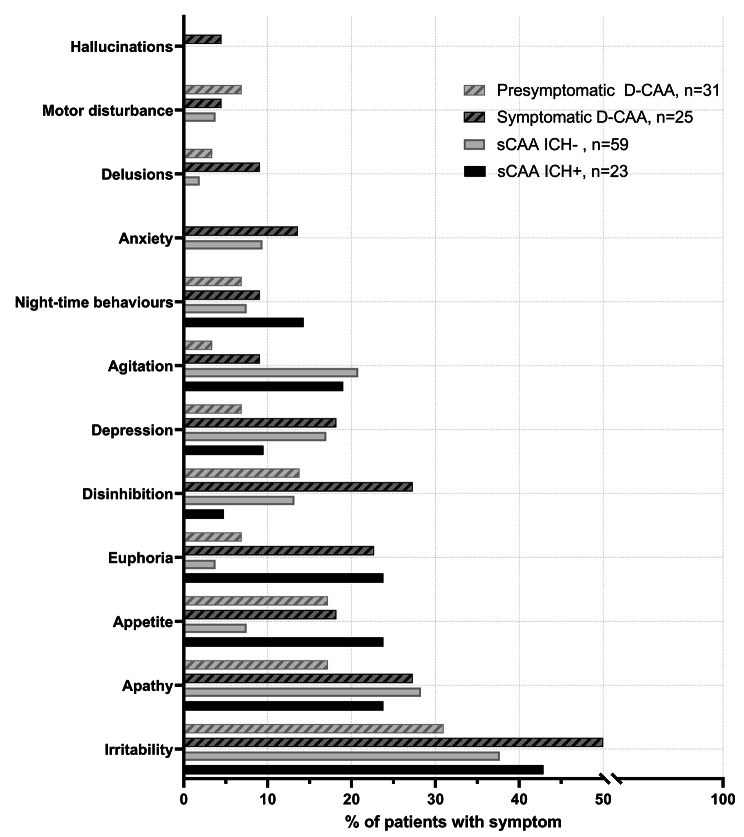
Neuropsychiatric symptom-profiles (informant-reported NPI-Q) in sporadic and Dutch-type cerebral amyloid angiopathy, stratified by previous ICH

We administered 166 NPI-Qs. Informants reported ≥ 1 NPS in 42(57%, 95%CI:45–67) patients with sCAA, 29(57%, 95%CI:43–69) with D-CAA, and 3(7%, 95%CI:3–19) controls (Fig. 1, Table S2). Across all patient groups, informants most frequently reported irritability (29[39%, 95%CI:28–51] sCAA, 20[39%, 95%CI:26–54] D-CAA) and apathy (20[27%, 95%CI:17–39] sCAA, 11[22%, 95%CI:11–35] D-CAA). The third most frequent symptom was agitation in sCAA (*n* = 15, 20%) and disinhibition in D-CAA (*n* = 10, 20%). All NPS except motor behaviors were reported at least as frequent in symptomatic as in presymptomatic D-CAA. Restricting to only NPS with moderate/severe severity, the NPS-profile remains similar (≥ 1 NPS in 31% of patients with sCAA; 33% with D-CAA; 7% controls; Table S3).

**Fig. 2:**
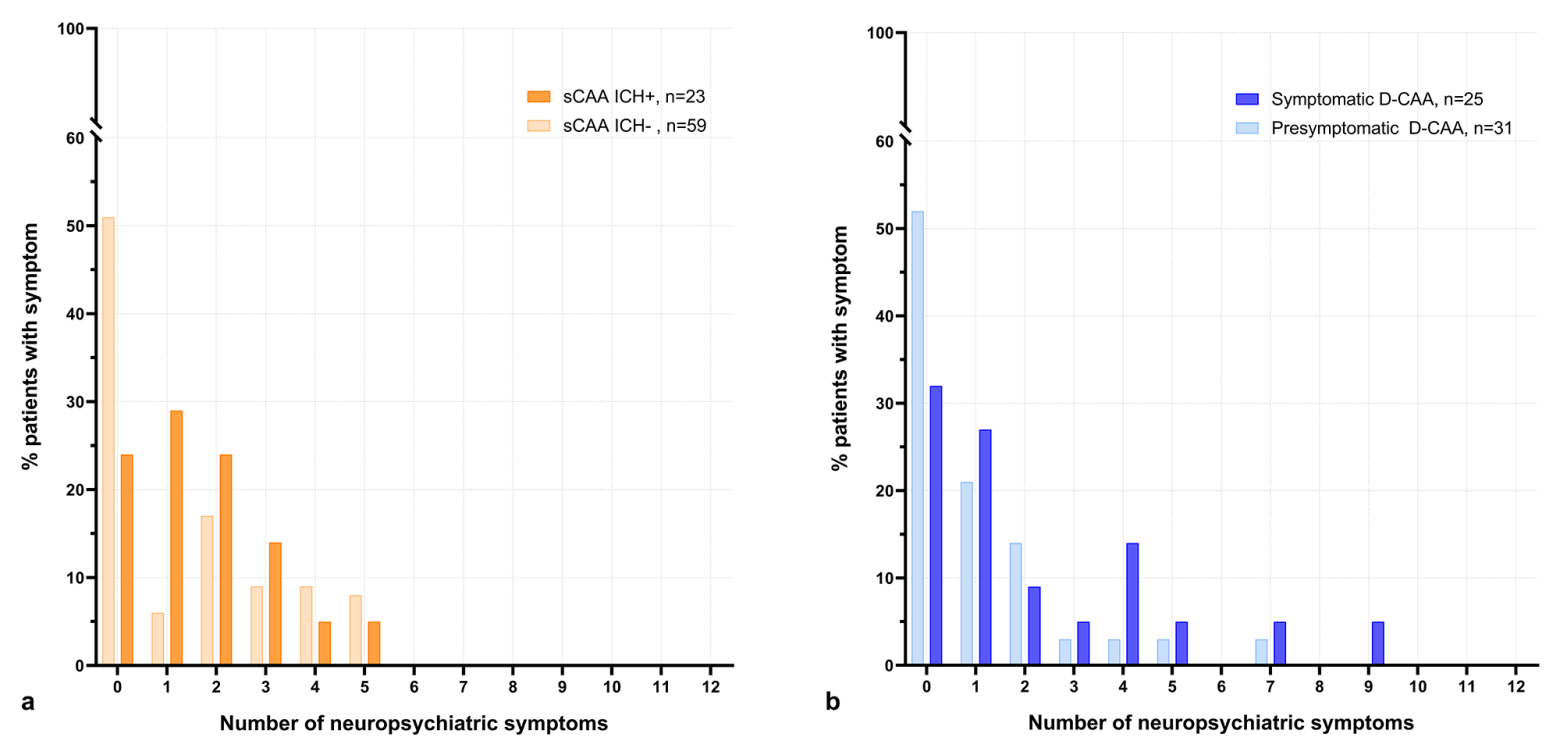
Neuropsychiatric symptom-counts (informant-reported NPI-Q) Legend: Comparisons between sporadic (**panel a**) and Dutch-type (**panel b**) cerebral amyloid angiopathy (sCAA, **panel a**), stratified by previous intracerebral hemorrhage (ICH)

Informants of patients with previous ICH reported NPS more frequently than those without (≥ 1 NPS:72% vs. 49%, Fig. 2). Patients with previous ICH generally had higher NPI-Q severity than those without (median: 1[0–5] vs. 0[0–3]; Table 2). Patients with presymptomatic D-CAA had higher odds of NPS than controls (OR:12[95%CI:3–47]). The NPI-Q total differed between the different CAA-groups and controls, but not between sCAA and D-CAA (sCAA-ICH + adj.β = 1.4[95%CI:0.6–2.3]; sCAA-ICH- 1.3[0.6-2.0]; symptomatic D-CAA 2.0[1.1–2.9]; presymptomatic D-CAA 1.2[0.1;2.2]).

**Table 2:** Prevalence of apathy and irritability in patients with CAA and controls

*n*	sCAA, all *82*	sCAA ICH + *23*	sCAA ICH- *59*	D-CAA, all *56*	D-CAA symptomatic *25*	D-CAA presymptomatic *31*	Control *43*
**NPI-Q**							
Administered, n(%)	74 (90)	21 (88)	53 (91)	51 (91)	22 (88)	29 (94)	41 (95)
Total, median[IQR]	1 [0,3]	1 [1,2]	0 [0,3]	1 [0,2]	1 [0,4]	0 [0,2]	0 [0,0]
Severity, median[IQR]	1 [0,3]	2 [0,3]	1 [0,3]	0 [0,3]	1 [0,5]	0 [0,2]	0 [0,0]
Caregiver burden if ≥ 1 NPS, median[IQR]	4 [3,6]	4 [2,6]	4 [3,9]	3 [2,8]	4 [2,9]	3 [2,3]	3 [2,4]
**Starkstein Apathy Scale**							
Administered, n(%)	74 (90)	21 (88)	53 (91)	53 (95)	24 (96)	29 (94)	42 (98)
Score, median[IQR]	12 [7,17]	12 [8,16]	11 [7,18]	12 [7,16]	11 [6,18]	12 [8,15]	6 [3,8]
Apathy present, score ≥ 14, n(%)*	12 (31)	5 (28)	7 (33)	19 (37)	10 (44)	9 (31)	1 (7)
**Irritability Scale**							
Administered, n(%)	42 (51)	19 (79)	23 (40)	52 (93)	23 (92)	29 (94)	15 (35)
Score, median[IQR]	9 [3,14]	8 [3,10]	10 [4,16]	8 [2,15]	8 [4,14]	5 [1,14]	6 [3,12]
Irritability present, score ≥ 14, n(%)*	12 (29)	4 (21)	8 (35)	14 (27)	6 (26)	8 (28)	4 (27)
**CES-D**							
Administered, n(%)	36 (44)	16 (67)	20 (34)	38 (68)	14 (56)	24 (77)	14 (32)
Depressed, score ≥ 16, n(%)*	17 (47)	8 (50)	9 (45)	18 (47)	6 (43)	12 (50)	1 (7)

CES-D Center for Epidemiological Studies Depression scale; D-CAA Dutch-type cerebral amyloid angiopathy; sCAA sporadic CAA; NPI-Q Neuropsychiatric Inventory Questionnaire, informant based

* Reported as % of administered questionnaire/interview

Apathy (169 SAS administered) was present in 12(31%) patients with sCAA, 19(37%) with D-CAA, and 1(7%) control. This was similar across all ICH-stratified groups (Table 2, Table S4, Figure S2). Congruency between SAS and NPI-Q was ≥ 80% (Appendix S2). The apathy score differed between disease groups and the controls, but not between sCAA and D-CAA (sCAA-ICH + adj.β = 6.5[95%CI:3.1–9.8]; sCAA-ICH- 6.3[3.7–8.9]; symptomatic D-CAA: 6.7[3.3–10.1]; presymptomatic D-CAA 7.2[3.0-11.4]). Patients with presymptomatic D-CAA had apathy reported more often than controls (OR:6.3, 95%CI:0.7–56). Overlap of apathy on the SAS, and depression on the CESD was limited (Appendix S2).

The relative frequency of irritability according to the Irritability Scale (109 administered) was similar across all groups (12[29%] sCAA, 14[27%] D-CAA, 4[27%] controls). Patients with presymptomatic D-CAA and controls had similar odds of having irritability (OR:1.1[95%CI:0.3–4.3]; Irritability Scale).

### The relationship of NPS with cognition

Cognitive performance scores and the restricted sensitivity analysis incorporating the SDMT in the processing speed domain Z-scores are shown in Table S5 and Appendix S3. Total NPI-Q score was associated with decreased processing speed (adj.β=-0.6[95%CI:-0.8;-0.4], *p* < 0.001) and with having CAA, particularly in those with history of ICH (sCAA-ICH + adj.β = 1.0[95%CI:0.1;1.9], sCAA-ICH- β = 0.6[-0.03;1.3], symptomatic D-CAA β = 1.5[0.7;2.3], presymptomatic D-CAA β = 0.7[-0.004;1.5]). Total NPI-Q score was associated with worse executive function (adj.β=-0.4[95%CI:-0.6;-0.1], *p* = 0.003), independent of disease group (sCAA-ICH + adj.β = 1.3[0.3;2.2]; sCAA-ICH + β = 1.0[0.4;1.7]; symptomatic D-CAA β = 1.6[-0.7;2.4]; presymptomatic D-CAA β = 1.0[0.2;1.8]). The restricted sensitivity analysis yielded a similar result (adj.β processing speed:-0.7[95%CI:-1.0;-0.4], *p* < 0.001).

### NPS and MRI CAA-burden

The MRI CAA-burden can be observed in Table 3. In patients with sCAA, 38% of ICHs were in the frontal lobe and 8% in the limbic structures of the brain; this was 30% and 23% in patients with D-CAA (Table 3). NPI-Q total score was not associated with CAA-burden on MRI (adj.β = 0.08[95%CI:-0.2;0.3], *p* = 0.5), after controlling for age, sex and disease group. NPI-Q total score was not associated with frontal cSS (adj.β = 0.06[95%CI:-0.7;0.8], *p* = 0.9) or frontal CMB (adj.β = 0.1[95%CI:-0.2;0.5], *p* = 0.4) after controlling for the same confounders.

**Table 3:** Cognitive profile and MRI CAA-burden score in patients and controls

*n*	sCAA, all *82*	sCAA ICH+ *23*	sCAA ICH- *59*	D-CAA, all *56*	D-CAA symptomatic *25*	D-CAA presymptomatic *37*	Controls *43*
Executive Function, Z-score* mean(SD)	-0.1 (1.2)	-0.3 (1.6)	-0.1 (1.1)	0.3 (1.2)	0.1 (1.3)	0.6 (1.0)	0.6 (0.7)
Processing speed, Z-score* mean(SD)	-0.8 (1.3)	-1.0 (1.5)	-0.7 (1.2)	-0.3 (1.2)	-0.6 (1.4)	-0.1 (0.9)	0.2 (0.7)
Memory, Z-score* mean(SD)	-0.7 (1.0)	-1.0 (1.1)	-0.6 (1.0)	-0.7 (0.9)	-1.1 (0.9)	-0.4 (0.8)	0.4 (0.8)
CAA-burden score, median[IQR]	4 [4,5]	4 [3,6]	4 [4,5]	4 [1,5]	4 [4,5]	1 [1,4]	1 [1,1]
Frontal lobe cSS, n(%)	47 (57)	12 (52)	35 (59)	14 (29)	10 (40)	4 (16)	0 (0)
Frontal lobe CMB, n(%)							
0	11 (13)	3 (16)	8 (16)	20 (25)	1 (4)	19 (76)	39 (91)
1–10	35 (43)	10 (53)	25 (51)	1 (2)	1 (4)	0 (0)	2 (5)
11–20	4 (5)	1 (5)	3 (6)	6 (11)	5 (20)	1 (4)	0 (0)
>20	18 (22)	5 (26)	13 (27)	21 (38)	16 (64)	5 (20)	0 (0)

cSS cortical superficial siderosis; CMB cerebral microbleeds; D-CAA Dutch-type cerebral amyloid angiopathy; sCAA sporadic CAA

* age, sex and education adjusted Z-scores

### Sex-differences

Men with CAA had higher odds of having any NPS than women (OR:2.8, 95%CI:1.3–5.8). There were no sex-differences for having of apathy (OR men vs. women:1.7; 95%CI:0.7–4.1; SAS) or irritability (OR:1.2; 95%CI:0.5–2.9; Irritability Scale).

### Apathy and Aβ burden in CSF

We analysed CSF-samples from 39 patients with sCAA (12 with NPI-Q based apathy) and 26 controls (all without apathy). Patients with and without NPI-Q based apathy had similar CSF-levels of Aβ-40 (median[IQR]: 7.6 [6.7–9.4] vs. 8.0 [6.5–9.6] ng/mL, *p* = 0.9), Aβ-42 (median[IQR]: 342 [277–410] vs. 373 [282–466] pg/mL, *p* = 0.7), total tau (median[IQR]: 432 [296–698] vs. 434 [312–512] pg/mL, *p* = 0.7) and phosphorylated tau (median[IQR]: 61.4 [45.8–94.6] vs. 56.0 [37.4–72.3], *p* = 0.4). Controls had higher Aβ-40 (median[IQR]: 12.5 [10.9–14.6] ng/mL) and Aβ-42 (median[IQR]: 1030 [813–1274] pg/mL) than patients, but similar total tau (median[IQR]: 356 [280–472] pg/mL) and phosphorylated tau (median[IQR]: 41.3 [33.9–60.9] pg/mL) levels.

### Sensitivity analyses

The following statements compare excluded patients (*n* = 52, all FOCAS/AURORA) to their included counterparts. Excluded patients with sCAA more often had previous ICH (13[52%] vs. 23[28%], Table S6). Excluded patients with D-CAA were older (mean age 46 years vs. 52 years). Excluded patients had Z:0.3 better memory. General cognition, executive functioning and processing speed were comparable for in- and excluded patients, but excluded controls had Z:0.5 better executive function and memory. Other baseline characteristics and cognition of the in- and excluded participants were similar.

## Discussion

In this study, informants reported neuropsychiatric symptoms in about one third to one half of patients with sCAA and D-CAA. Apathy and irritability were present in one third of CAA-patients. Apathy was associated with sCAA and D-CAA, but results for irritability were inconclusive. Our data suggest that NPS might be an early clinical sign of D-CAA. Having more NPS was associated with decreased processing speed and executive functioning. We found no association between NPS-count and overall or frontal CAA disease burden on MRI. Men had NPS more often than women, but there were no clear sex-differences for apathy or irritability.

Patients with symptomatic D-CAA and sCAA had a highly similar neuropsychiatric profile, with increased frequency and severity of NPS in patients with a history of ICH or cognitive decline. The four most frequently reported NPS in sCAA (irritability, apathy, agitation and depression) in our study resembled a previous study, albeit with lower prevalence estimates of agitation and depression [7]. The near absence of hallucinations, delusions and motor disturbances also resonates with the literature.

The apathy frequency of 30–37%, measured by the SAS, resembled previous findings in CAA-literature, where 35% of 43 patients with sCAA had NPI-Q based apathy [7]. Our estimate was also in line with the results of another study (*n* = 37, 43% apathetic according to the SAS) in patients with sCAA without previous ICH [8]. We note that informants might confuse apathy with depression. However, while this was not assessed in regression analysis for statistical efficiency, the overlap of apathy and depression on the NPI-Q, or on the CESD and SAS was limited. Considering the similarities between our findings in sCAA and the previous literature, we expect that our informant-based findings are robust across all patient groups.

Approximately one third of patients had high scores for irritability on the Irritability Scale and NPI-Q. In sCAA, this is slightly less than reported in previous literature (37%, NPI-Q based) [7]. In controls, the prevalence of irritability was similar to CAA on the Irritability Scale, but lower on the NPI-Q. As the Irritability Scale more extensively measures the underlying construct than the NPI-Q, we expect the true proportion of control participants with irritability to be approximately one third, suggesting that there was no clear difference between patients and controls. We note that this might be due to measurement properties of the irritability scale, or because relatively fewer irritability scales than NPI-Qs were analyzed. Thus our results for irritability remain inconclusive.

We observed higher odds of having NPS, but not specifically of apathy or irritability, in presymptomatic D-CAA, compared with controls. We note that patients with presymptomatic D-CAA were younger than controls, but also that their odds were similar to the other CAA-groups. No previous literature for D-CAA was available for comparison. However, previous studies have identified WMH to be correlated with development of NPS in CSVD patients, possibly as a consequence of damage to reward systems and structures related to decision making [39–41]. As non-hemorrhagic tissue injury is thought to be one of the early MRI markers in D-CAA to become abnormal, this might explain the onset of NPS in the presymptomatic phase of D-CAA [42]. Genetic disease-related psychological factors might also explain part of the NPS in (pre)symptomatic D-CAA.

We found that total NPS-count was associated with decreased processing speed and executive function. Interpreting these results is challenging. While in AD both domains were previously associated with total NPS-count, a previous CAA-study did not observe this association [7, 39, 43]. Our discrepancy with the CAA-literature might be due the use of different neuropsychological instruments. However, our sensitivity analysis yielded comparable results, despite adding another instrument and restricting sample size. Another explanation might be that answering strategies, such as speed-accuracy trade-off for errorless performance, are not captured in quantitative analysis of neuropsychological tests [44, 45]. This increases the influence of sampling error and random variation when comparing studies. Information to assess this trade-off was unavailable in our data. Further, differences in executive function between in- and excluded participants might have caused effect-underestimation, but might also be attributed to random variation. Overall, our findings suggest that NPS may be associated with processing speed and executive function, but our results should be interpreted with caution.

In our study, NPS and total (or frontal) CAA-burden score on MRI were not associated. This was unexpected, as we hypothesized that accumulation of vascular damage amounts to disruption of regulating circuits (such as but not limited to the limbic system, the reward systems, or those related to the orbitofrontal cortex) and cognitive decline, thereby causing NPS. Moreover, in patients with ICH, apathy and hyperactivity were previously associated with a CAA MRI-profile [9]. Our findings align with one previous sCAA study, but contrasts with another, in which high SAS-score correlated with increased CAA-burden and with disruption of white matter tracts on diffusion tensor imaging [7, 8]. Unfortunately, we were not able to assess white matter volume or microstructural integrity. However, our result might be a consequence of using the CAA-burden score, a measure that gives less weight to ischemic burden, and where different patterns of cSS, CMB, WMH and CSO-EPVS can accumulate to the same score. The same is true for the frontal burden, which only comprised hemorrhagic lesions (presence/absence of cSS and number of CMBs). This might have been an insufficiently sensitive measure for quantifying the frontal burden, which might explain the lack of an association with NPS. Future research might best focus on incorporating non-hemorrhagic burden with quantitative measures, and microstructural integrity to study this further.

In our study, men seemed to be more prone to have NPS than women, despite similar age profiles. This contrasts a recent meta-analysis in AD-patients (50% NPI-Q based) that did not find sex-differences for NPS, apathy or irritability, although men exhibited more severe apathy [5]. In a previous CAA-study, the NPS-incidence was similar between sexes [7]. Thus it remains unclear if our finding is generalizable. Nevertheless, clinicians should be aware that sex-differences in NPS may exist, as these symptoms can sometimes be treatable and have implications for patient and caregiver education.

A strength of our study is that we were able to study symptomatology, cognition and sex-differences in a patient sample that equals the combined previous literature in size. We confirmed findings for patients with sCAA without a history of ICH and added new knowledge about those with previous ICH. Also, we were able to investigate NPS in a unique hereditary cohort of patients with D-CAA, a relatively pure form of CAA, in both the presymptomatic and symptomatic phases. Further, by using the same instruments as in the previous literature, but also using more extensive validated questionnaires, we were able to study apathy and irritability in more depth, while retaining comparability and reliability despite using data from different source cohorts.

Our study has other limitations. First, we measured NPS rather than clinical (neuro)psychiatric diagnoses: the questionnaires do not differentiate between clinical and non-clinical complaints [3]. Additionally, NPS were only evaluated by informants, which might differ from clinical ratings. Therefore, our results should be interpreted as informant-reported symptomatology rather than as clinical diagnoses. Second, our questionnaire study is inherently susceptible to motived-responder bias. Specifically, informant responses might be biased in those with higher caregiver burden. Unfortunately, no reference group with patients with non-neurologic disease was available for comparison. Replication could assess this. Third, the controls are a selected sample. However, we believe that this group provides a useful reference for the NPS-profile in absence of CAA, or (subjective) cognitive decline. Fourth, data was collected from separate source cohorts and thus is susceptible for systematic differences. However, the neuropsychological testing protocols and study assessments of the source cohorts were designed to be comparable, thereby minimizing impact on the generalizability of our findings. Fifth, the CAA-burden score is a simplified compound score that quantifies the CAA-lesion load by combining multiple rating scales. Due to its ceiling effects (i.e., for cSS and WMH quantity, or number of CMBs), the loss of information by categorization, and its inherent omission of other (micro)structural lesions, the clinical applicability of our burden-related findings was restricted. In extension, unfortunately data on cortical thinning of the different lobes was not available. As cortical thinning might be associated with apathy, future research might investigate this further for patients with CAA [46]. Finally, because we considered depression and anxiety best measured by patient-reporting and no previous NPS-related CAA-literature existed at study conception, we did not collect data on these symptoms or assess them in-depth in our informant-based study.

In conclusion, neuropsychiatric symptoms, specifically apathy, are common in patients with sCAA and D-CAA. These symptoms are already present before the occurrence of ICH and men might be more susceptible to developing them. Worse cognition, but not evidently CAA MRI-burden score, may be associated with having more NPS. Neurologists should inform patients and caregivers of these disease consequences and treat or refer patients who are burdened by these symptoms appropriately.

### Electronic supplementary material

Below is the link to the electronic supplementary material.

Supplementary Material 1

## Data Availability

No datasets were generated or analysed during the current study.
