# Supplementary material for: Neuropsychiatric symptoms with focus on apathy and irritability in sporadic and hereditary cerebral amyloid angiopathy
Source: Alzheimers Res Ther. 2024 Apr 6;16:74. doi: 10.1186/s13195-024-01445-4 (PMC10998371; doi:10.1186/s13195-024-01445-4)
Supplement: Supplementary file 1 — Supplementary Material 1 [file 13195_2024_1445_MOESM1_ESM.docx]

**SUPPLEMENTAL MATERIAL**

**Supplemental Methods**

*Participants*

The CAFE controls had no history of brain- or neurodegenerative disease if they had no history of major brain pathology, such as spontaneous parenchymal ICH, ischemic stroke, neurodegenerative disease, brain tumours, -infection or inflammation. Intact global cognition in cognitive screening was defined as Modified Telephone Interview for Cognitive Status ≥35 or Mini Mental State Examination ≥28.^16,17^

The FOCAS controls were recruited in the context of a sub-study on blood-brain barrier imaging with contrast enhancement. Within this context, but not in the context of the current investigation, they were age- and sex-matched to patients enrolled in the sub-study.

The informants were the partner (84%), child (9%), or other (7%) of the patient or control. *Clinical data collection*

Symptomatic ICH was defined as a presentation to the hospital due to focal neurological complaints with a corresponding lobar ICH on CT or MRI. History of self-reported apathy was obtained from the corresponding study visit (AURORA/FOCAS participants only).

*Neuropsychiatric questionnaires*

The Starkstein Apathy Scale for measuring apathetic symptoms was developed and validated with good internal validity in patients with Parkinson’s disease and AD, and has been used in various types of research, such as in community dwelling elderly.^4^

*MRI Data Collection and Analysis*

MRI of all AURORA/FOCAS participants were collected on the same 3.0T scanner (Philips Healthcare, Best, the Netherlands). MRI of all BIONIC participants were collected at the same 3.0T scanner (Siemens Health engineers, PrismaFit; Erlangen, Germany) In case of discrepancies between the two independent raters, an experienced neuroradiologist was consulted.

ICH, cSS and CMB were assessed on susceptibility-weighted imaging (SWI). We recorded presence, number, and location of ICH and CMB. We classified cortical superficial siderosis (cSS) as absent, focal (restricted to ≤3 sulci), or disseminated (>3 sulci involved), and recorded its presence/absence in the frontal lobe. cSS contiguous or anatomically correlated to ICH were not included in the focal/disseminated cSS categories. CSO-EPVS were assessed on T2-weighted MRI using a validated 4-point visual rating scale (0= no EPVS; 2= 11-20 EPVS; 3= 21-40 EPVS; 4= >40 EPVS). White matter hyperintensities were assessed on fluid-attenuated inversion recovery imaging (FLAIR) using the Fazekas rating scale (range 0-3 for DWMH, range 0-3 for PVH). The CAA-burden score summarizes lobar CMBs (2-4: 1 point; ≥5: 2 points), cSS (focal: 1 point, disseminated: 2 points), WMH (DWMH Fazekas ≥2 or PVH Fazekas 3: 1 point) and CSO-EPVS (grade 3-4: 1 point) to a 6-point ordinal scale.

cSS was rated by Van der Plas and Kaushik; WMH by Koemans and Kaushik (AURORA/FOCAS first visit) and by Van Dort and Van der Plas (AURORA/FOCAS second visit); CSO-EPVS by Koemans, Kaushik and Van der Plas; CMB and ICH by Koemans (AURORA/FOCAS first visit), Van der Plas and Van der Zwet (AURORA/FOCAS second visit). In case of disagreement, AURORA/FOCAS scans were discussed with an experienced neuroradiologist at the LUMC. BIONIC MRI data was assessed by De Kort and Van Berckel-Smit. In case of disagreement, BIONIC scans were discussed with Schreuder.

*Cerebrospinal fluid analysis*

In all source cohorts, participants were invited to undergo a lumbar puncture according to the local protocol. CSF was collected in polypropylene tubes, centrifuged, aliquoted, and stored in polypropylene tubes at 80°C. All CSF analyses were performed at the RUMC. To avoid bias, samples of patients and controls were randomly analysed. CSF Aβ40, Aβ42, tau phosphorylated and total tau levels were quantified using the Lumipulse chemiluminescent immunoassay (Fujirebio, Ghent, Belgium). The samples were analysed in different batches; however, we adhered to strict guidelines under the ISO15189 guidance to control that inter-assay variation is kept within predefined limits of variation for each assay. Participants in whom CSF was not collected (e.g., due to refusal of lumbar puncture), were excluded from this analysis.

*Handling of missing data*

If no 3T MRI was present at the same time as, or within 12 months before neuropsychiatric questionnaire administration, MRI-markers were inferred (n=2 subjects) as: absent when absent at subsequent study visit; maximum if maximum at previous study visit; or as missing (n=2) if neither absent nor maximum at adjacent visits. There were n=4 subjects in whom MRI could not be performed due to safety issues, n=2 due to claustrophobia, and n=3 due to cohort protocol.

Patients who did not provide consent for specific study procedures (e.g., neuropsychological testing or lumbar puncture) were excluded for that analysis.

*Regression analysis*

Model assumptions for all regression models were checked and not violated. In the regression model for frontal CAA burden, the values of frontal CMBs were truncated at 100, where all values >100 were categorized as ‘100+’.

**Figure S1:** Flowchart of inclusions


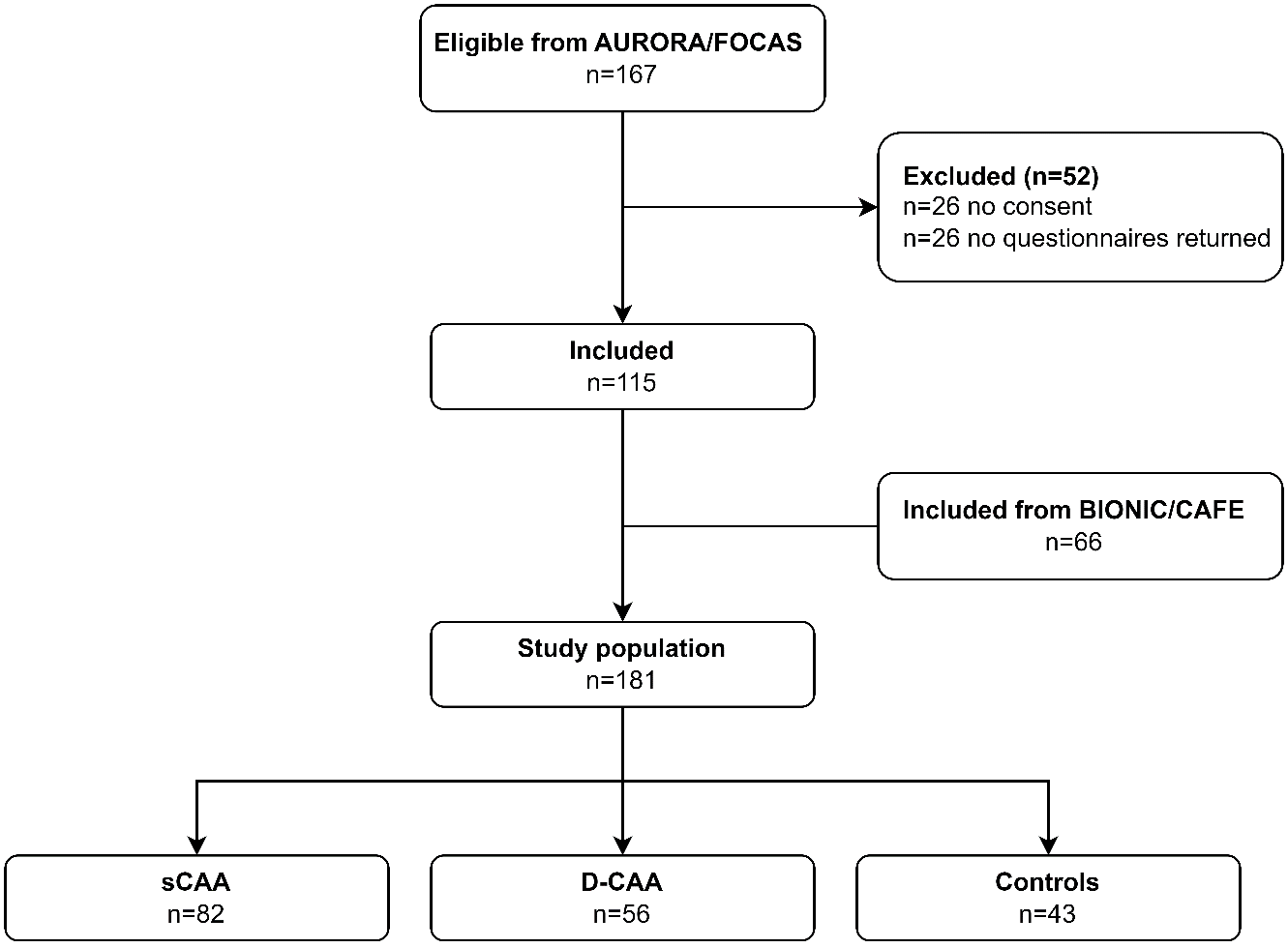


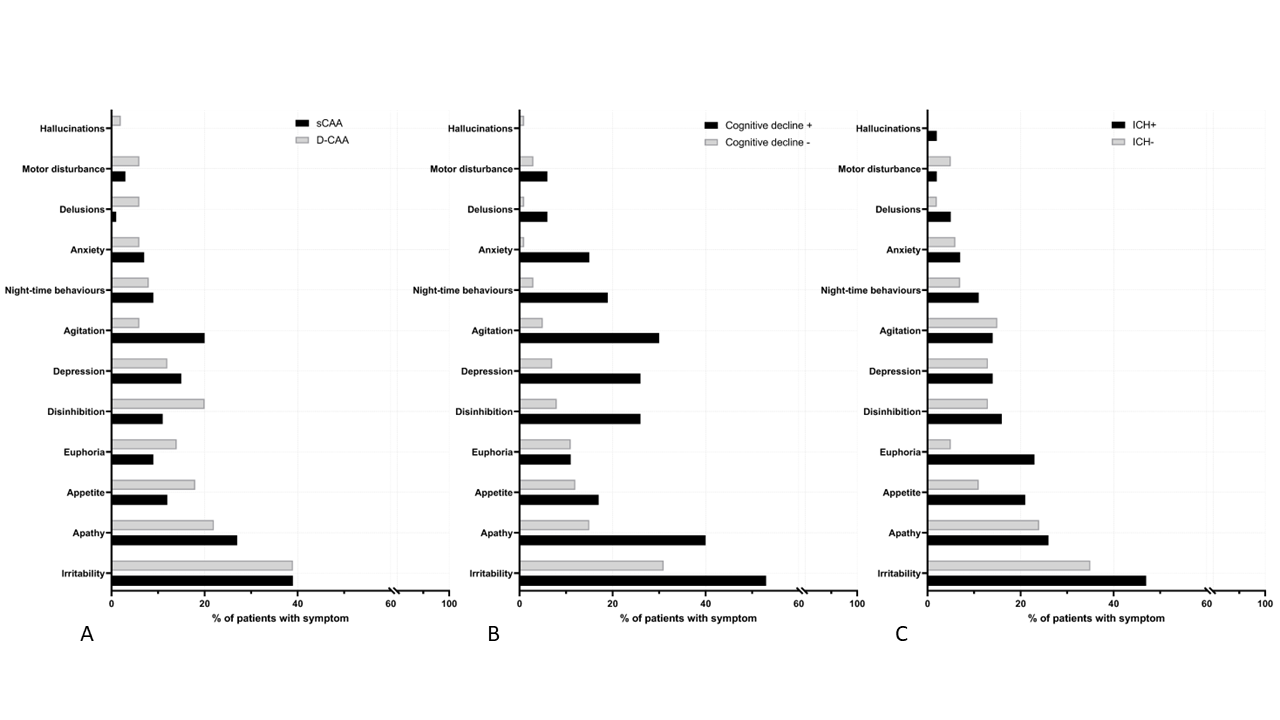
**Figure S2:** NPS profile of patients with CAA, stratified by CAA-subtype (A), cognitive decline (B; MoCA<26) or history of ICH (C)

**Table S1:** CAA-related MRI-markers in patients and controls

|  | **sCAA, all** | **D-CAA, all** | | **Controls** | |
| --- | --- | --- | --- | --- | --- |
| N(%) | *82* | *56* | | *43* | |
| WMH Fazekas ≥2 | 44 (54) | 25 (45) | | 9(21) | |
| CSO-EPVS grade ≥3 | 38 (46) | 41 (73) |  | 7 (16) |  |
| CMBs |  |  |  |  |  |
| 0 | 2 (2) | 13 (23) |  | 38 (88) |  |
| 1-10 | 23 (28) | 5 (9) |  | 3 (7) |  |
| 10-20 | 10 (12) | 1 (2) |  | - |  |
| >20 | 42 (51) | 30 (54) |  | - |  |
| cSS |  |  |  |  |  |
| Focal | 12 (15) | 10 (18) |  | - |  |
| Disseminated | 38 (46) | 6 (11) |  | - |  |
| CMB cerebral microbleed CSO-EVPS centrum semi-ovale enlarged perivascular spaces; cSS cortical superficial siderosis; D-CAA Dutch-type cerebral amyloid angiopathy; sCAA sporadic cerebral amyloid angiopathy; WMH White matter hyperintensities; | | | | | |

### **Table S2:** Neuropsychiatric symptoms (informant-based) compared between sporadic cerebral amyloid angiopathy (sCAA), Dutch-type CAA, and controls.

|  | **sCAA, all** | sCAA ICH+ | | | sCAA ICH- | | **D-CAA, all** | | D-CAA symptomatic | | D-CAA presymptomatic | | **Controls** | |
| --- | --- | --- | --- | --- | --- | --- | --- | --- | --- | --- | --- | --- | --- | --- |
| N(%, *95%CI*)* | *82* | *23* | | | *59* | | *56* | | *25* | | *31* | | *43* | |
| ≥1 NPS reported | 42 (57, *45-68*) | 16 (76) | | | 26 (49) | | 29 (52, *42-70*) | | 15 (68) | | 14 (48) | | 3 (7, 2*-21*) | |
| Agitation | 15 (20, *12-32*) |  | 4 (18) |  | 11 (20) |  | 3 (6, *2-*17) |  | 2 (9) |  | 1 (3) |  | 0 |  |
| Anxiety | 5 (7, *3-16*) |  | - |  | 5 (9) |  | 3 (6, *2-17*) |  | 3 (14) |  | - |  | 1 (2, *0-14*) |  |
| Apathy | 20 (27, *18-39*) |  | 5 (24) |  | 15 (27) |  | 11 (22, *12-36*) |  | 6 (27) |  | 5 (17) |  | 0 |  |
| Appetite | 9 (12, *6-22*) |  | 5 (24) |  | 4 (7) |  | 9 (18, *9-31*) |  | 4 (18) |  | 5 (17) |  | 0 |  |
| Delusions | 1 (1, *0-8*) |  | - |  | 1 (2) |  | 3 (6, *2-17*) |  | 2 (9) |  | 1 (3) |  | 0 |  |
| Depression/ Dysphoria | 11 (15, *8-25*) |  | 2 (10) |  | 9 (16) |  | 6 (12, *5-25*) |  | 4 (18) |  | 2 (7) |  | 2 (5, 0*-17*) |  |
| Disinhibition | 8 (11, *5-21*) |  | 1 (5) |  | 7 (13) |  | 10 (20, *10-34*) |  | 6 (27) |  | 4 (14) |  | 0 |  |
| Euphoria/ Elation | 7 (9, *4-19*) |  | 5 (24) |  | 2 (4) |  | 7 (14, *6-27*) |  | 5 (23) |  | 2 (7) |  | 0 |  |
| Hallucinations | 0 |  | 0 |  | 0 |  | 1 (2, *0-10*) |  | 1 (5) |  | 0 |  | 0 |  |
| Irritability/ Lability | 29 (39, *28-51*) |  | 9 (43) |  | 20 (36) |  | 20 (39, *26-54*) |  | 11 (50) |  | 9 (31) |  | 2 (5, *1-18*) |  |
| Motor disturbances | 2 (3, *0-10*) |  | - |  | 2 (4) |  | 3 (6, *2-17*) |  | 1 (5) |  | 2 (7) |  | 0 |  |
| Night-time behaviours | 7 (9, *4-19*) |  | 3 (15) |  | 4 (7) |  | 4 (8, *3-20*) |  | 2 (9) |  | 2 (7) |  | 0 |  |
| D-CAA Dutch-type cerebral amyloid angiopathy; sCAA sporadic cerebral amyloid angiopathy; ICH intracerebral haemorrhage  *Reported as % of administered questionnaire: sCAA ICH+ n=21, ICH- n=53; D-CAA symptomatic n= 22, pre-symptomatic n=29; control= 41 | | | | | | | | | | | | | | |

### **Table S3:** Neuropsychiatric symptoms (informant-based) compared between sporadic cerebral amyloid angiopathy (sCAA), Dutch-type CAA, and controls, restricted to ‘moderate/severe’ severity

|  | **sCAA, all** | **D-CAA, all** | | **Controls** | |
| --- | --- | --- | --- | --- | --- |
| N(%, 95%CI)* | *82* | *56* | | *43* | |
| ≥1 NPS reported | 23 (31, *21-43*) | 17 (33, *21-48*) | | 3 (7, *3-19*) | |
| Agitation | 4 (5) | 2 (4) |  | - |  |
| Anxiety | 3 (4) | 1 (2) |  | - |  |
| Apathy | 11 (15) | 5 (10) |  | - |  |
| Appetite | 3 (5) | 4 (8) |  | - |  |
| Delusions | 1 (4) | 1 (2) |  | - |  |
| Depression/ Dysphoria | 4 (15) | 5 (10) |  | - |  |
| Disinhibition | 1 (4) | 4 (8) |  | - |  |
| Euphoria/ Elation | 2 (1) | 2 (4) |  | - |  |
| Hallucinations | - | - |  | - |  |
| Irritability/ Lability | 9 (12) | 9 (18) |  | 3 (7) |  |
| Motor disturbances | 1 (1) | - |  | - |  |
| Night-time behaviours | 2 (3) | 3 (6) |  | - |  |
| D-CAA Dutch-type cerebral amyloid angiopathy; sCAA sporadic cerebral amyloid angiopathy; ICH intracerebral haemorrhage  *Reported as % of administered questionnaire: sCAA ICH+ n=22, ICH- n=52; D-CAA symptomatic n= 22, pre-symptomatic n=29; control= 41 | | | | | |

|  | **ICH+** | **ICH-** | **Cognitive decline +** | **Cognitive decline -** |
| --- | --- | --- | --- | --- |
| N(%, 95%CI)* | *48* | *90* | *52* | *82* |
| ≥1 NPS reported | 31 (73, *57-85*) | 34 (46, *35-57*) | 35 (74, *59-86*) | 33 (45, *33-57*) |
| Agitation | 6 (14, *6-29*) | 12 (15, *8-25*) | 14 (30, *18-45*) | 4 (5*, 2-14*) |
| Anxiety | 3 (7, *2-20*) | 5 (6, *2-14*) | 7 (15, *7-29*) | 1 (1, *1-8*) |
| Apathy | 11 (26, *14-41*) | 20 (24, *16-35*) | 19 (40, *27-56*) | 11 (15, *8-25*) |
| Appetite | 9 (21, *11-36*) | 9 (11, *5-20*) | 8 (17, *8-31*) | 9 (12, *6-22*) |
| Delusions | 2 (5, *1-17*) | 2 (2, *0-9*) | 3 (6, *2-19*) | 1 (1, *1-8*) |
| Depression/ Dysphoria | 6 (14, *6-29*) | 11 (13, *7-23*) | 12 (26, *14-41*) | 5 (7, *3-16*) |
| Disinhibition | 7 (16, *7-31*) | 11 (13, *7-23*) | 12 (26, *14-41*) | 6 (8, *3-17*) |
| Euphoria/ Elation | 10 (23, *12-38*) | 4 (5, *2-13*) | 5 (11, *4-24*) | 8 (11, *5-21*) |
| Hallucinations | 1 (2, *0-14*) | 0 | 0 | 1 (1, *1-8*) |
| Irritability/ Lability | 20 (47, *31-62*) | 29 (35, *25-47*) | 25 (53, *38-68*) | 23 (31, *21-43*) |
| Motor disturbances | 1 (2, *0-14*) | 4 (5, *2-13*) | 3 (6, *2-19*) | 2 (3, *0-10*) |
| Night-time behaviours | 5 (11, *4-25*) | 6 (7, *3-16*) | 9 (19, *10-34*) | 2 (3, *0-10*) |
|  |  |  |  |  |
| Apathy on Starkstein Apathy scale | 15 (35) | 16 (20) | 15 (32) | 14 (19) |
| Irritability on irritability scale | 10 (23) | 16 (20) | 13 (28) | 12 (16) |
| ICH intracerebral haemorrhage; Cognitive decline based on MoCA;  *Reported as % of administered questionnaire, all patients with CAA combined;  n=43 respondents ICH+, n=82 ICH-, abnormal cognition=47, normal cognition n=74 | | | | |

### **Table S4:** Neuropsychiatric profile of patients with CAA combined, stratified by history of ICH, or cognitive decline

### **Table S5:** Raw scores of cognitive testing of included patients

|  | **sCAA, all** | sCAA ICH+ | sCAA ICH- | **D-CAA, all** | D-CAA symptomatic | D-CAA presymptomatic | **Controls** |
| --- | --- | --- | --- | --- | --- | --- | --- |
| n | *82* | *23* | *59* | *56* | *25* | *37* | *43* |
| **Executive Function, Z-score mean(SD)** | **-0.1(1.2)** | **-0.3(1.6)** | **-0.1(1.1)** | **0.3(1.2)** | **0.1(1.3)** | **0.6(1.0)** | **0.6(0.7)** |
| Cognitive flexibility (TMT B/A), median[IQR] | 2.7 [2.1;3.5] | 3.0 [2.2-3.4] | 2.7[2.1-3.5] | 2.2 [1.9-3.3] | 2.3 [1.9-3.5] | 2.1 [1.9-2.8] | 2.5 [2.0-2.9] |
| TMT-B, seconds, median[IQR] | 117 [74,174] | 139 [83,182] | 104[73,171] | 65 [48,109] | 80 [61,145] | 52 [39,78] | 76 [58,90] |
| Stroop interference, seconds, median[IQR] | 100 [75,129] | 92 [70,147] | 100[76,129] | 62 [58,74] | 72 [63,88] | 58 [50,62] | 69 [58,75] |
| Stroop III, seconds, median[IQR] | 131 [104,188] | 142 [108,208] | 131[104,175] | 89 [82,106] | 104 [93,115] | 84 [76,90] | 95 [82,103] |
| FAB*, median[IQR] | 17 [15,18] | 17 [15,18] | 16[14,17] | 18 [17,18] | 17 [15,18] | 18 [18,18] | 17 [16,18] |
| **Processing speed, Z-score mean(SD)** | **-0.8 (1.3)** | **-1.0 (1.5)** | **-0.7 (1.2)** | **-0.3 (1.2)** | **-0.6 (1.4)** | **-0.1 (0.9)** | **0.2 (0.7)** |
| TMT-A, seconds, median[IQR] | 40 [28,62] | 40 [28,61] | 40 [30,62] | 32 [22,40] | 39 [31,44] | 23 [17,32] | 29 [24,39] |
| Category fluency, words, median[IQR] | 20 [15,23] | 20 [15,23] | 20 [15,23] | 22 [17,27] | 19 [16,27] | 23 [21,29] | 25 [20,28] |
| Stroop I, seconds, median[IQR] | 55 [47,64] | 62 [53,64] | 54 [46,63] | 48 [44,60] | 50 [46,7] | 45 [43,53] | 46 [40,52] |
| Stroop II, seconds, median[IQR] | 74 [62,90] | 81 [71,91] | 70 [59,89] | 58 [54,67] | 62 [55,82] | 56 [52,60] | 59 [52,63] |
| SDMT*, median[IQR] | 35 [21,47] | 34 [26,40] | 35 [21,48] | 39 [39,39] | 39 [39,39] | - | 47 [41,52] |
| **Memory, Z-score mean(SD)** | **-0.7 (1.0)** | **-1.0 (1.1)** | **-0.6 (1.0)** | **-0.7 (0.9)** | **-1.1 (0.9)** | **-0.4 (0.8)** | **0.4 (0.8)** |
| D-CAA Dutch-type cerebral amyloid angiopathy; IQR interquartile range; sCAA sporadic cerebral amyloid angiopathy; SD standard deviation; SDMT: symbol digit modalities test; TMT-A/B trail making test A/B; FAB frontal assessment battery;  *SDMT administered in n=38 sCAA, n=1 D-CAA, n=27 controls. FAB administered in n=38 sCAA, n=49 D-CAA, n=16 controls  Executive function, processing speed and memory are displayed as age, sex and education adjusted Z-scores | | | | | | | |

### **Table S6:** Characteristics of patients who did not fill out questionnaires

|  | **sCAA, all** | sCAA ICH+ | sCAA ICH- | **D-CAA, all** | D-CAA symptomatic | D-CAA presymptomatic | **Control** |
| --- | --- | --- | --- | --- | --- | --- | --- |
| n | *25* | *13* | *12* | *15* | *5* | *10* | *12* |
| Age, mean (SD), years | 71 (8) | 71 (8) | 71 (8) | 46 (14) | 62 (5) | 38 (8) | 70 (13) |
| Men, n(%) | 17 (68) | 8 (62) | 9 (75) | 6 (40) | 3 (60) | 3 (30) | 7 (58) |
| Educational level, n(%) |  |  |  |  |  |  |  |
| Low | 6 (24) | 3 (23) | 3 (25) | 3 (20) | 2 (40) | 1 (10) | 3 (25) |
| Average | 5 (20) | 3 (23) | 2 (17) | 7 (47) | 1 (20) | 6 (60) | 3 (25) |
| High | 5 (20) | 4 (31) | 1 (8) | 5 (33) | 2 (40) | 3 (30) | 3 (25) |
| Medical history, n(%) |  |  |  |  |  |  |  |
| Symptomatic ICH | 13 (52) | 13 (100) | - | 5 (33) | 5 (100) | - | - |
| Depression | 2 (8) | 1 (8) | 1 (8) | - | - | - | - |
| Previous symptomatic ICH count, median [IQR] | 0 [0, 1] | 1 [1, 1] | - | 0 [0, 1] | 1 [1, 1] | - | - |
| Self-reported apathy, n(%) | 7 (28) | 4 (31) | 3 (25) | - | - | - | 2 (17) |
| MoCA, median [IQR] | 24 [24,26] | 24 [24,25] | 23 [22,26] | 28 [26,29] | 28 [25, 29] | 28 [26, 29] | 27 [25,28] |
| Executive functioning, Z-score, median [IQR] | -0.3 [-2.3; 0.2] | -0.4 [-2.0; 0.1] | -0.3 [-2.6; 0.3] | 0.4 [-0.2; 0.7] | -0.2 [-0.6; 0.1] | 0.4 [0.0, 0.8] | 1.1 [0.8, 2.0] |
| Processing speed, Z-score, median [IQR] | -0.9 [-1.5; -0.3] | -0.9 [-1.3; -0.2] | -0.9 [-2.2; -0.3] | 0.1 [-0.8; 0.5] | -0.9 [-1.1; -0.8] | 0.2 [-0.5; 0.6] | 0.5 [-0.2; 0.8] |
| Memory, Z-score, median [IQR] | -1.1 [-1.5; -0.8 | -1.1 [-1.3; -0.5] | -1.1 [-2.0; -1.0] | -0.4 [-1.2; 0.2] | -0.8 [NA] | 0.1 [-1.1; 0.3] | 0.9 [0.6, 1.0] |
| D-CAA: Dutch-type cerebral amyloid angiopathy, sCAA: sporadic CAA, MoCA: Montreal Cognitive assessment, ICH: intracerebral haemorrhage, IQR: interquartile range, SD: standard deviation Data of excluded patients were collected from the first study visit in their source cohort. Data is displayed as available. In contrast to the manuscript, cognition Z-scores are displayed as median[IQR] rather than mean(SD) due to paucity of data. Reason for exclusion from the current study: n=26 no informed consent provided for receiving questionnaires; n=26 non-responder after sending questionnaire | | | | | | | |

**Appendix S1 – Unadjusted regression models**

In the univariate model, the NPI-Q total score was associated with the disease group (unadjusted β[95%CI] sCAA ICH+ 1.5[0.6-2.4]; sCAA ICH- 1.3[0.7-2.0]; D-CAA symptomatic: 2.0[1.1-2.8]; D-CAA presymptomatic 1.0[0.2-1.8], controls as reference).

The apathy score was associated with the disease group (unadjusted β sCAA ICH+ 6.7[3.3-10.0]; sCAA ICH- 6.3 [3.8-9.0]; D-CAA symptomatic 6.2[3.0-9.5]; D-CAA presymptomatic 6.0[2.9-9.0], controls as reference).

The NPI-Q total score was associated with executive function (unadjusted β -0.4[-0.6;-0.1]) and disease group (sCAA ICH+ 1.2[0.3-2.1]; sCAA ICH- 1.0[0.4-1.7]; D-CAA symptomatic 1.5[0.7-2.4]; D-CAA presymptomatic 1.0[0.2-1.8], controls as reference).

The NPI-Q total score was associated with processing speed (unadjusted β -0.6[-0.8;-0.4]) and having ICH in patients with CAA (sCAA ICH+ 0.8[-0.01; 1.7]; sCAA ICH- 0.6[-0.03;1.3]; D-CAA symptomatic 1.4[0.6-2.2]; D-CAA presymptomatic 0.7[-0.004;1.5], controls as reference).

The NPI-Q total score was not associated with CAA-burden (unadjusted β 0.1[-0.1; 0.3]) separate from disease type (sCAA ICH+ 1.2[-0.01; 2.3]; sCAA ICH- 1.0[-0.1; 2.0], D-CAA symptomatic 1.7[0.5-2.9], D-CAA presymptomatic 1.0[0.1-1.9], controls as reference).

**Appendix S2: Congruency of informant-based and self-reported apathy, irritability and depression**

Nine (21%) patients with sCAA, 10(19%) with D-CAA and 0 controls self-reported apathy during their study visit (collected from FOCAS/AURORA only). In patients who had apathy according to the NPI-Q, 3/9 (33%) of those with sCAA and 3/11 (27%) with D-CAA also self-reported apathy. In those with SAS-reported apathy, 5/12 (42%) with sCAA and 4/19 (21%) with D-CAA had congruent self-reporting. In the sCAA group, there were 32/38 (84%) congruent SAS and NPI-Qs (7 with apathy on both), with 2 participants only having SAS-reported apathy and 4 only having NPI-Q reported apathy. In D-CAA there were 40/50 (80%) participants with congruent SAS and NPI-Q (10 with apathy on both), 1 with only NPI-Q reported apathy and 9 with SAS-reported apathy. In excluded patients, self-reported apathy was less frequent than in included patients (none vs 10[19%].

Of the 60(33%) AURORA/FOCAS participants who self-reported personality changes, 17(59%) of patients with sCAA and 13(42%) of patients with D-CAA reported irritability, whereas no controls self-reported irritability. In the sCAA group, there were 30/40 (75%) a congruent irritability scale and NPI-Qs (9 with irritability on both), with 3 participants only having irritability on the irritability scale, and 7 only NPI-Q reported irritability. In D-CAA there were 38/51 (75%) participants with a congruent irritability scale and NPI-Q (10 with irritability on both), 9 with only NPI-Q reported irritability and 4 with only irritability scale-reported irritability.

The following table shows congruency of informant-reported apathy and self-reported depression. This was not entered in the regression models due to reduced statistical power of the model and to limit the number of statistical tests in our investigation.

|  | Apathy+ | Apathy- | Irritability+ | Irritability- |
| --- | --- | --- | --- | --- |
| **sCAA, n(%)** |  |  |  |  |
| Depression+ | 8 (24) | 7 (21) | 8(23) | 9(11) |
| Depression- | 4 (12) | 14 (42) | 2(2) | 16(46) |
| **D-CAA, n(%)** |  |  |  |  |
| Depression+ | 5 (14) | 11 (31) | 4(11) | 13(15) |
| Depression- | 6 (17) | 14 (39) | 6(7) | 13(36) |
| **Controls, n(%)** |  |  |  |  |
| Depression+ | 0 (0) | 1 (7) | 0(0) | 1(7) |
| Depression- | 1 (7) | 12 (86) | 4(29) | 9(64) |
| *Depression: CESD ≥16; Apathy: SAS≥14; Irritability: Irritability scale ≥14* | | | | |

**Appendix S3: Cognitive performance of patients with sCAA, patients with D-CAA and controls**

Z-score -3SD was assigned for 6 aborted tests (1 aborted the Stroop-III, 5 aborted the TMT-B, 1 aborted both tests). These tests were all aborted after being started due to the patient being unable to understand or complete the test, and/or due to patient frustration with a wish to abort the test, all unrelated to impairments of vision or motor function.

Overall, patients with sCAA (10% cognitive impairment, 7% below average) performed similar to patients with D-CAA on executive functioning (10% impairment, 2% below average) and worse than controls (0% below average). Similarly, processing speed was worse in sCAA (24% impairment, 16% below average) than in D-CAA (14% impairment, 12% below average) and controls (0% below average).
